# The Use of Sports Rehabilitation Robotics to Assist in the Recovery of Physical Abilities in Elderly Patients with Degenerative Diseases: A Literature Review

**DOI:** 10.3390/healthcare11030326

**Published:** 2023-01-21

**Authors:** Fangyuan Ju, Yujie Wang, Bin Xie, Yunxuan Mi, Mengyun Zhao, Junwei Cao

**Affiliations:** 1 Department of Physical Education, Yangzhou University, Yangzhou 225012, China; 2 Department of Business, Yangzhou University, Yangzhou 225012, China

**Keywords:** rehabilitation robot, assistive technology sports rehabilitation, continuation therapy, degenerative diseases, elder

## Abstract

The increase in the number of elderly patients with degenerative diseases has brought additional medical and financial pressures, which are adding to the burden on society. The development of sports rehabilitation robotics (SRR) is becoming increasingly sophisticated at the technical level of its application; however, few studies have analyzed how it works and how effective it is in aiding rehabilitation, and fewer individualized exercise rehabilitation programs have been developed for elderly patients. The purpose of this study was to analyze the working methods and the effects of different types of SRR and then to suggest the feasibility of applying SRR to enhance the physical abilities of elderly patients with degenerative diseases. The researcher’s team searched 633 English-language journal articles, which had been published over the past five years, and they selected 38 of them for a narrative literature review. Our summary found the following: (1) The current types of SRR are generally classified as end-effector robots, smart walkers, intelligent robotic rollators, and exoskeleton robots—exoskeleton robots were found to be the most widely used. (2) The current working methods include assistant tools as the main intermediaries—i.e., robots assist patients to participate; patients as the main intermediaries—i.e., patients dominate the assistant tools to participate; and sensors as the intermediaries—i.e., myoelectric-driven robots promote patient participation. (3) Better recovery was perceived for elderly patients when using SRR than is generally achieved through the traditional single-movement recovery methods, especially in strength, balance, endurance, and coordination. However, there was no significant improvement in their speed or agility after using SRR.

## 1. Introduction

Currently, the global population is aging. In many countries, life expectancy has increased to 70 years or more. For the first time in history, in 2020, the number of people aged 60 or older exceeded the number of children under five years old, globally. Furthermore, over the coming decades, the greatest increase in aging will occur in developing countries [1]. As one of the largest developing countries, China’s adoption of the family planning policy as a basic state policy in 1982 has affected the country’s demographic structure and has aggravated the aging problem. The current fertility of the young people in the country has also changed; therefore, low birth rates and low mortality rates have gradually become the main reasons for the aging of Chinese society. According to the projections of the United Nations Development Program (UNDP), the population of those over 65 years old in China will reach 334 million by 2050, and the China Aging Study 2022 has predicted that it will peak at 425 million in 2057. In the future, China will be the country with the largest elderly population in the world and will be facing the serious challenge of its population aging.

The excessive aging of the population will lead to various social problems, such as a lack of economic growth, overburdened public finances, and a sharp increase in the cost of medical care, with medical care becoming the main social burden. As elderly people age further, their physiological functions deteriorate and their physical abilities decline, to some degree [2]. Physical ability is the most basic motor skill necessary for an individual’s daily life and labor to be maintained, and it can be classified as strength, endurance, balance, coordination, agility, and speed, according to its nature. In addition, the elderly population is more susceptible to various degenerative diseases, such as Parkinson’s disease (PD), stroke, multiple sclerosis (MS), etc. The probability that elderly people will suffer from degenerative diseases will also gradually increase as they continue to age [3,4,5]. Degenerative diseases not only damage the physical and mental health of the elderly and reduce their quality of life, but they also increase the medical expenses of their families and society. Due to the declining physical ability of the elderly, the process of motor rehabilitation usually requires a significant amount of time and a conducive environment to achieve the desired levels of rehabilitation; however, there is a shortage of rehabilitation nursing staff who are able to provide round-the-clock care, thus, the cost of such rehabilitation is high. This expense and scarcity of care has led to further significant challenges for China’s health and social systems in meeting the rehabilitation needs of its elderly population [6,7]. Consequently, the demand for sports rehabilitation robots in the medical rehabilitation field is increasing. ”Sport” rehabilitation robots is a rehabilitation robot that can provide motor rehabilitation assistance (including providing assistance or guidance) to patients. Sport rehabilitation robots can help patients with degenerative diseases recover their physical abilities, improve their immune system and metabolic activity [8], return to normal life, and reduce the financial strain on their families due to rehabilitation. Furthermore, the use of sports rehabilitation robots can reduce the burden on rehabilitation instructors, enable data detection during training, and assist in rehabilitation in a controlled and repeatable manner in order to complete quantitative assessments [9]. Previous studies have found that most of the research in this field has focused only on the design and development of devices for medical rehabilitation robots. Furthermore, many of these studies have focused on studying medical rehabilitation robots’ role in the physical recovery of PD and stroke patients. However, fewer articles have investigated the use of sports rehabilitation robots in assisting in the recovery of the physical abilities of elderly patients with degenerative diseases.

Most of the previous review papers have been organized and analyzed for the types of rehabilitation robots or their working principles. The novelty of this review is to analyze and compare how different rehabilitation robots work and how effective they are in restoring the physical abilities of elderly patients with degenerative diseases, focusing on the ways in which locomotion-based rehabilitation robots help patients to restore their physical abilities and their effectiveness.

This paper compiles and analyzes the studies related to the high level of motor rehabilitation robots that are being used to promote physical activity in the elderly; compares the advantages and shortcomings of different types of motor rehabilitation robots, in terms of their working methods and their effects; and then proposes feasible suggestions for the application of motor rehabilitation robots in enhancing the physical abilities of elderly patients with degenerative diseases.

## 2. Materials and Methods

The scope and review of the literature search followed the EQUATOR guidelines, which improve the quality and transparency of research. We ensured that articles related to rehabilitation robots were searched for rather than articles related to rehabilitation and robots, separately. Five databases were searched using the PRSIMA for Protocols guidelines: (1) Web of Science; (2) Engineering Village; (3) Science Direct; (4) the online library, Wiley; and (5) Scopus.

The search was conducted using a Boolean logic combination search for keywords, including the following: ‘rehabilitation robotics’, ‘physical activity, ‘human–robot interaction’, ‘rehabilitation training’, ‘robotic therapy’, ‘assistive technology’, and ‘older people’.

First, a total of 633 articles were obtained from the literature search across the five databases, published over the last five years (2018–2022). In total, 23 duplicate articles were deleted; 134 articles with non-relevant articles were excluded; and 6 articles that could not be downloaded through various sources were deleted. A total of 470 articles were obtained after this screening.

**Figure 1:**
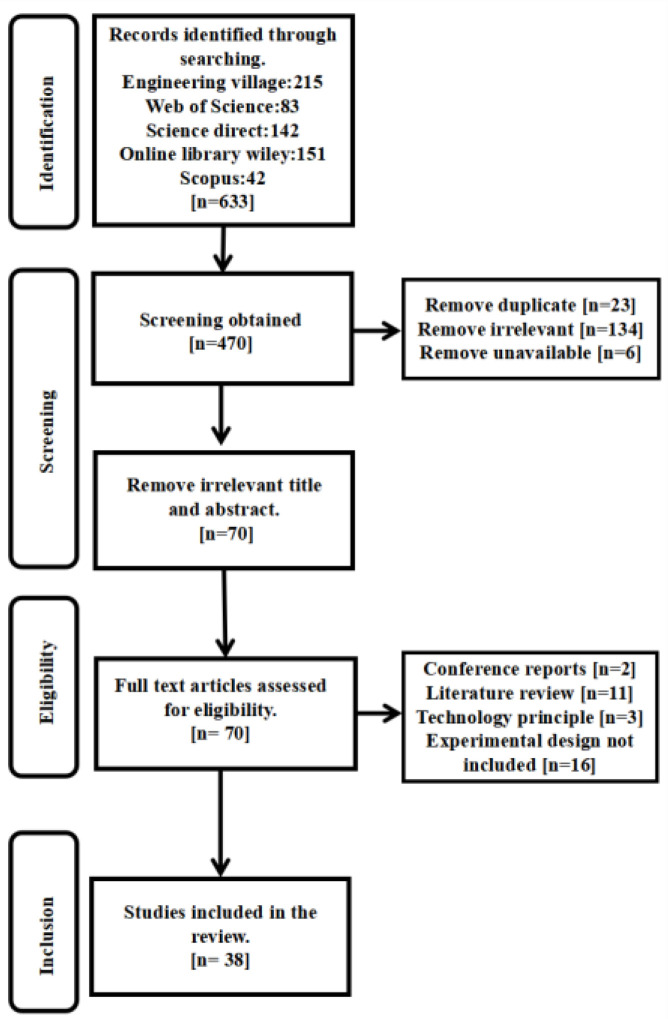
Flowchart detailing the systematic search, screening, eligibility, and inclusion procedure.

Second, 70 of these articles were read in full. They were chosen out of the 469 articles by screening the titles and abstracts. The last five researchers further screened the remaining 70 articles by reading the full text of the articles separately, and the articles that included conference reports, literature reviews, the introduction of a technology principal, and those that did not contain an experimental design, were excluded. The screening flow chart is shown in Figure 1.

When disagreements arose, a sixth author participated in the discussion, until a consensus was reached among them. At the end of this process, 38 qualitatively published articles (Table 1) were eventually included in the synthesis for a narrative review.

## 3. The Value of Robot-Assisted Technology in the Motor Rehabilitation of the Elderly

In the context of global aging, the risk of diseases in the elderly increases as their age advances; therefore, there is a growing demand for robot-assisted motor rehabilitation technology in society. Robot-assisted rehabilitation has, thus, become a prominent research topic, with broad application prospects [10]. Current assistive technologies that utilize robotics include mobility devices (wheelchairs, prosthetics, and external skeletons); specialized assistive devices (visual, auditory, and voice communication); and assistance in accessing information technology and peripherals for people with disabilities.

Most of the communication between devices and users is currently achieved through human–computer interaction systems; however, current human–computer interaction methods usually only have one of these functions, and only the elderly communicated instructions to the robot, meaning that there is no way to obtain feedback from the environment and to adapt to the behavior of the elderly user, accordingly [48]. The design and development of sports rehabilitation robots is not a simple combination of equipment and technology, but it also involves neuroscience; sports biomechanics; sports human body science; ergonomics; robotics, automation, and control; and other professional field knowledge, making it a typical multidisciplinary intersection of complex systems.

Since the operating object of the sports rehabilitation robot is a human, its performance must meet the requirements of adaptability to individual differences and environmental changes, fluency of human-machine interaction, safety in the face of abnormal situations, and adaptability to human physiology and psychology, thus putting higher requirements on the accuracy, reliability, and intelligence level of the control system.

Therefore, the current system of sports rehabilitation robots integrates research fields—such as artificial intelligence, human–computer interactions, and machine learning technology—to achieve intelligent, humanized, and accurate rehabilitation assistance [49]. Elderly patients who are suffering from degenerative diseases—such as Parkinson’s disease, stroke, and multiple sclerosis—account for a large number of the users of motor rehabilitation robots. These patients mainly present with upper and lower limb dysfunction, trunk weakness, decreased proprioception, decreased balance and postural control, gait abnormalities, and abnormal movement patterns [11]. Patients with multiple sclerosis also commonly experience impaired mobility and mobility limitations, which are caused by a combination of several factors—such as increased susceptibility to muscle fatigue, pain, abnormal tone, and falls [50].

Compared with the traditional manual, assisted rehabilitation training, robot-assisted motor rehabilitation and neurological rehabilitation training has unique advantages. Firstly, sports rehabilitation robots can provide high-intensity and repeatable rehabilitation training. Once popularized, they will be able to greatly relieve the pressure on the level of staff input in rehabilitation institutions and will be able to reduce the workload of clinical rehabilitation physicians. However, a more prominent advantage is that robots can provide flexible and precise rehabilitation training, which can enhance the effectiveness of patients’ rehabilitation. The main example of this is that the rehabilitation robot can combine modern multimedia and interactive technology in patients’ training to stimulate their interest and enthusiasm in participating in the rehabilitation training, and to mobilize their awareness and ability to actively participate in the training, which, thus, promotes the recovery and compensation of patients’ neurological functions [12].

At the same time, the sports rehabilitation robot can also combine sensor technology with multiple modalities to accurately detect changes in a patient’s physical condition in real-time and then use these data to adjust rehabilitation training strategies [51]. Research on motor rehabilitation robots is important for relieving the pressure on medical resources and rehabilitation manpower investments, and for improving the effectiveness of elderly patients’ rehabilitation training.

## 4. Working Methods and the Effects of the Assistance of Different Types of Sports Rehabilitation Robots

Degenerative diseases can have an impact on the physical abilities of strength, agility, balance, endurance, coordination, and speed for elderly patients. In particular, they can lead to limb dysfunction and to the inability to perform basic physical activities. Different types of sports rehabilitation robots have emerged in the market to assist with the dysfunction of different parts of the human body. These robots meet the different rehabilitation needs of patients through their range of different working methods to help restore limb functions and to return the patient to normal life.

### 4.1. Classification and Characteristics of Sports Rehabilitation Robots

**Figure 2:**
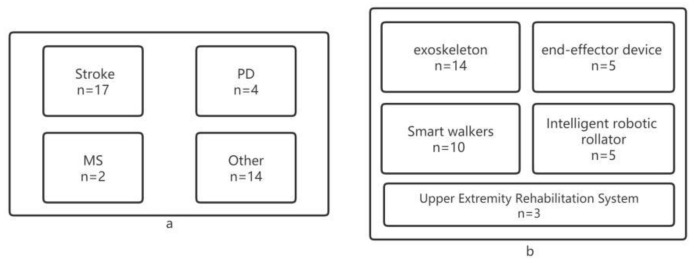
(**a**) Number of literature accounted for by different diseases; (**b**) number of literature accounted for by different types of rehabilitation robots.

According to the results of our literature analysis and according to previous classification methods (Figure 2), we found that the sports rehabilitation robots are currently classified into the following groups: upper extremity rehabilitation system, end-effector robot, smart walker, intelligent robotic rollator, and robotic exoskeletons. These classifications help them to respond to the rehabilitation needs of patients with different diseases.

#### 4.1.1. Upper Extremity Rehabilitation System: Helps Patients Recover Upper Extremity Function

Among the robots used for upper limb function recovery is the QM-FOrMS, a portable and cost-effective upper limb rehabilitation system. The system is able to give some exercise instruction to help patients perform active upper extremity activities; however, it only provides a small amount of assisted strength, which is a limitation for patients with low levels of physical ability [13].

This system, first proposed by Lin (2016), advocates the acquisition of EMG signals from the patient’s upper limb muscle groups to identify their motor intent and drive them to active training in order to complete the training task, through a combination of electrical stimulation and robot-assisted technology [52]. It is evident that the upper extremity rehabilitation system has a role in enhancing patients’ initiative. Moreover, the integrated sports intervention approach, which combines different assistive technologies, has a different training focus and, thus, has different training advantages. Due to the complex structure of the upper limb, the restoration of the structural function of the upper limb requires comprehensive consideration of the functional characteristics of each part of the shoulder, elbow joint, wrist, and hand. Therefore, Lin’s (2019) study modified and pointed out that the upper limb recovery system is characterized by its open and novel approach to recovery, and that the system’s unique training feedback system would further increase patients’ motivation and participation [13,53], by allowing them to actively engage in diverse training, based on light cues. Ultimately, Lin argued that this would lead to a holistic recovery of the patient’s upper limb function.

#### 4.1.2. End-Effector Robot Promotes the Range of Motion and Flexibility of the Patient’s Hand Joints

The end-effector robot uses assistive devices to assist in the movement of more than 20 free flexible joints in the hand, which helps patients to record the recovery process of their small muscles and nerves, and helps them, to a certain extent, to achieve dexterity and coordination in their hand functions. For example, REO-GO includes a telescopic arm and a screen, and offers different movement therapy modalities, according to the patient’s motor ability and motivation. This robot has two types of handles, spherical and platform, and places EMG electrodes on the patient’s upper limbs to record their muscle activity [11]. In a literature collection analysis, the scholar Moggio (2022) noted that end-effector rehabilitation robots can assist patients with stretching and flexion movements within a range of motions close to that of normal human fingers and can generate enough assisted force at the fingertips to provide a boost to the patient’s rehabilitation [52]. In addition, Fuzzy Sliding Mode Control technology was developed for functional finger rehabilitation. It provides the possibility of rehabilitating each phalanx individually which is very important in the finger rehabilitation process [14,54,55]. VR technology is also gradually being incorporated into the design of rehabilitation robots [56]. Other studies have also pointed out that the recovery of the arm needs to take into account, not only the direction of movement, but also the recovery of the nervous system. In this complexity of movement and motor control, rehabilitation can provide a guide for motor recovery that influences the neurobiology of neuronal plasticity, by providing controlled, repetitive, and variable patterns [57]. Thus, the end-effector robot can promote a range of motions and flexibility in a patient’s hand joints; however, its application in restoring the neurological system of the hand is more limited and less impressive.

#### 4.1.3. Smart Walkers Enhance Leg Muscle Strength and Balance

Unlike the two types of robots discussed above (the upper limb rehabilitation system and the end-effector robot), the smart walker is a lightweight robotic assistive device. It is intended as a lower limb function recovery robot. It can provide support and assistance to patients and can improve their autonomy and the effectiveness of their rehabilitation work. In addition, it also has a certain degree of safety and does not restrict the range of motion in the patient’s joints or their amplitude, which allows the patients maximum freedom [15]. For elderly patients, leg strength and body balance are the basis for participating in rehabilitation activities. Sierra (2019) pointed out that the smart walker is able to select rehabilitation exercises of different intensities, which are based on the patient’s own recovery. This enables them to gain a better understanding of their recovery and to adapt more effectively to real walking conditions [16]. The smart walker can help patients to complete physical activities through assistive devices, can provide personalized exercise programs, and can give different levels of support and assistance to patients with different dysfunctions, which can play an active and effective role in lower limb muscle strength and balance functions [15,16,17]. Enhancing leg strength and body balance not only improves the ability of the elderly patients to live, but also improves their bodies’ neurological connections and speeds up their physical recovery. The smart walker can also help patients to perform rehabilitation exercises for different physical difficulties, can develop new rehabilitation programs, and can provide real-time assessments through built-in sensors, which improves patients’ basic mobility [18], thus, helping them to restore their normal ability levels and to improve their quality of life.

#### 4.1.4. Intelligent Robotic Rollator Integrally Enhances Physical and Cognitive Abilities

The intelligent robotic rollator, e.g., i-Walk, is an integrated set of sensing, navigation, and user–robot interaction modules, which are designed in such a way as to enable the real-time operation to support the envisioned user-assisted functions [19]. They are suitable for providing cognitive and walking assistance to people with mild to moderate motor impairments (e.g., the elderly), and they can combine user-adaptive motion control, navigation in dynamic environments, and cognitive assistance. In addition, they can provide stable human posture support, walking assistance, navigation in indoor and outdoor environments, health monitoring, and more [58,59].

The researchers believe that the greatest advantage of this type of robot is its high intelligence—it not only has a verbal human–computer interaction system, but also recognizes the patient’s commands and feeds the language back into the system to recognize and respond to the patient’s intentions. It breaks through the limitations of the support provided by assistive devices and takes the patient as the main intermediary by incorporating the patient’s intentions into a functional rehabilitation training program in order to achieve a holistic improvement in the patient’s physical and cognitive abilities.

#### 4.1.5. Robotic Exoskeletons Provide Site-Specific Muscle Training

The research on this topic generally agrees that robotic exoskeletons are one of the most-used motor rehabilitation robots in the rehabilitation field today [60]. Through their bionic design, they connect the human-like mechanical structure’s design with the patient, forming an integrated, wearable mobile device, which is driven by an external power source to alleviate movement disorders. Therefore, they achieve the dual purpose of sports rehabilitation and physical function recovery, by effectively providing a second skeleton for the patients [61].

The research in and the development of the technology for exoskeleton rehabilitation robots are currently more mature than they are in other areas. Consequently, there are not only local joint rehabilitation assistance robots, but also full-body, wearable exoskeleton robots. This connects to the individual patient in a wearable way and has multiple points, and, because its joint axis matches human joints, it can control the movement of all the patient’s joints and can therefore train muscles in specific areas during the rehabilitation training. Furthermore, it can also provide corresponding training for patients with limb dysfunction to help restore the working ability of their limbs [62]. At present, robotic exoskeletons can be divided into the following four types, according to where they are worn: upper limb robotic exoskeletons [20,21,22,23,24], lower limb robotic exoskeletons [25,26,27,28,29,30], whole body robotic exoskeletons [15], and ground robotic exoskeletons [31,32].

Compared with the first four types of robot-assisted technology discussed in this paper, the current challenges in robotic exoskeleton research and development are as follows: (1) its rigid structure and multi-link design affects the freedom of movement in a patient’s joints—a patient can only execute the angle and speed that has been fixed for their training method; (2) spending a long time repeating single action exercises could easily trigger other parts of a sports injury and, thus, the function of other parts of the body could be weakened; and (3) it is a non-intelligent functional system, which is unable to follow up a patient’s rehabilitation progress in a timely manner or adjust the movement strategy.

### 4.2. How the Sports Rehabilitation Robot Works

The working methods of motor rehabilitation robots can be divided into three categories: assistant tools as the main intermediaries, the patient as the main intermediary, and the sensors as the intermediaries.

The robots in which the assistant tools are the main intermediaries focus on stimulating the patient’s motivation for rehabilitation and on guiding the patient to carry out their rehabilitation training. The robots in which the patient is the main intermediary are mainly controlled by the patient’s will and they help the patient to complete their rehabilitation training movements. In the robots in which the sensors are the main intermediaries, myoelectric sensors automatically identify the patient’s rehabilitation needs and make the rehabilitation process more intelligent. Patients can choose the right exercise rehabilitation robot, according to their needs and to the different stages of their rehabilitation to improve its efficiency.

#### 4.2.1. Assistant Tools as the Main Intermediary: Robotic-Assisted Patient Participation in Motor Rehabilitation

Assistant tools, as the main type of rehabilitation robot, consist of software and hardware tools and generally include not only the robot, but also a rehabilitation system, which work in conjunction with each other. Because there are different types of assistive rehabilitation, the robots’ components have different characteristics, and their working methods vary accordingly. Usually, each component is directed towards the device’s function to guide the patient through their motor rehabilitation.

As described above (Section 4.1.1), the upper extremity rehabilitation system, QM-FOrMS, consists of a smart pad, a smart canister, and a mobile device, and it directs a patient’s arm movements through LED cues on the smart pad to guide them towards completing their rehabilitation movements [53]. In the FELXO-Arm1 rehabilitation system, this upper limb-assisted rehabilitation robot (ULRR), has multiple degrees of freedom, which enables it to provide a full range of assistance to the patient. It has an encoder and sensors attached to it to enable it to record the patient’s rehabilitation status. Furthermore, additional power units act on the patient’s elbow and shoulder joints. Information on the forces interacting between the patient and the robot is obtained through sensors mounted at the joint locations.

Using this information, the rehabilitation robot actively constructs an inverse dynamic model to precisely calculate and control the starting friction, motion friction, and motion compensation force of the patient’s affected limb, laying the foundation for the subsequent development of the treatment plan [33]. Moreover, together with the TOT rehabilitation theory, different virtual reality game training tasks are set to guide patients towards completing the established rehabilitation training program, which can effectively improve stroke symptoms in elderly patients [34]. In contrast, the above two types of assistive device-based robots work in such a way that the patient must undergo rehabilitation training according to the rehabilitation program that is set by the robot and that is inherent in it.

This type of approach can restore the medical staff’s motor rehabilitation purpose to a greater extent and does not negatively affect the final rehabilitation of the patient if the they choose to make a change in the rehabilitation’s movement trajectory, or if they decide to abandon the program due to the patient’s pre-control or insufficient motivation. The FELXO-Arm1 rehabilitation system and the ULRR upper limb assisted robot can personalize the activity parameters according to patients’ different needs and abilities, for example, by including the patients’ background complexity, the running speed, the training time, the background music. These factors can effectively cultivate patients’ motivation for active rehabilitation and can therefore enhance the rehabilitation results.

#### 4.2.2. Patient as the Main Intermediary: Patients Operate Assistive Devices for Motor Rehabilitation Activities

The patient-oriented motor rehabilitation robot is mainly patient-controlled, and the rehabilitation robot is manipulated to perform rehabilitation activities according to the patient’s wishes, and with the help of the robot’s functions. For example, the REO-GO upper limb rehabilitation robot first uses a platform to stabilize the patient’s upper limb, and then relies on the patient to actively apply grip force to the handle, after which the robot assists the patient in achieving the free movement of their upper limb [11]. Similarly, patients can use the HAL-SJ, a wearable exoskeleton robot, to actively assist in their training, by using the muscles’ action potentials, which are detected from the patients’ muscle fibers. This approach can help elderly patients to improve their knee’s mobility and its synergistic contraction. Furthermore, this rehabilitation training method can reduce antagonist musculature and synergistic muscle injuries [18].

The whole-body exoskeleton robot, FB-AXO, connects the upper and lower systems through the lumbar and spine modules. Patients with muscle weakness can remain standing with the external assistance provided by the whole-body exoskeleton robot and they can therefore actively operate the robot to complete the physical activities set in the rehabilitation training program. Ultimately, this enables them to achieve the purpose of their rehabilitation [35].

The motion rehabilitation robot mainly targets elderly patients and can help them with their personalized physical activity exercises, under the active operation of the patient. These robots can supplement the patient’s subjective rehabilitation and the corresponding parts of their rehabilitation treatment. They can also allow the patient to perceive the recovery of their corresponding body parts through the feedback that they provide. The patients’ choice of an appropriate physical activity program (a choice offered by these robots) is also important for their subsequent rehabilitation. Therefore, this patient-led rehabilitation robot only provides external assistance, which maximizes the patient’s initiative in the rehabilitation process, unlike the robot-led rehabilitation approach, which requires patients to passively receive their rehabilitation treatment [32].

#### 4.2.3. Sensors as the Intermediaries: Myoelectric-Driven Robot Promotes Patient Participation in Sports Rehabilitation

Robots in which the sensors are the intermediaries have a significant impact on the way that the motor rehabilitation robot acts [36]. In addition, they contribute, to a certain extent, to the motor rehabilitation of elderly patients. Usually, upper limb exoskeleton robots (Rehab-Robotics) use myoelectric actuation as the mediating mode of action, and bioelectric sensors, such as EMG sensors, can detect the patient’s voluntary muscle activation in real-time and trigger the robot-assisted movements [63]. The lower extremity rehabilitation robot (Keeogo) places sensors on the thighs, knees, and calves of the elderly patients and uses a wearable design, which connects the calves to the thighs and suspends them from a lumbar carrier system, enabling the elderly patient’s hips to rotate freely and enabling the sensors to transmit data to a terminal for analysis during the rehabilitation exercises. This allows the robot to set up a rehabilitation medical program for them [37]. Ing-Jr Ding and Yu-Jui Chang had confirmed after research, the Kinect-sensor-based sport instructor robot was beneficial to rehabilitation and exercise training of the elderly. A GAD scheme for enhancing Kinect-sensor-based gesture recognition was proposed. In addition, three different types of state machine for formulating certain rehabilitation exercises in the sport instructor expert system were also presented [64].

With the sensors as the intermediaries, the data obtained by using EMG to observe the actual recovery of the patients will be more quantitative and objective than the data obtained through the observation of medical personnel and through personal perception. The use of a myoelectric drive to control the rehabilitation robot enables the patient to execute the rehabilitation movements precisely and enables the maximum possible fit with the patient’s rehabilitation wishes. Moreover, it also perfectly demonstrates the effectiveness of the medical rehabilitation program. Of course, how best to use myoelectric-driven rehabilitation robots to assist elderly patients with precise limb rehabilitation activities will become a major research focus in the future.

### 4.3. The Effect of the Sports Rehabilitation Robot

Traditional exercise rehabilitation programs are designed to focus on the characteristics of the patient’s disease and do not pay enough attention to the physical and mental characteristics and to the individual differences of the elderly patients [65]. For frail elderly patients, there are many barriers to exercise, including their physical inability to support their own physical activity; negative attitudes towards exercise; and their lack of sufficient exercise confidence [66]. In investigating the effectiveness of the existing motor rehabilitation robots, sports rehabilitation robots were found to be more effective in the treatment of degenerative diseases in the elderly, compared to the traditional rehabilitation methods, especially in secondary medical problems, such as osteoporosis, cardiovascular disease, respiratory problems, and intestinal dysfunction. Seniors who recover with the assistance of a sports rehabilitation robot also exhibit good behavioral characteristics and psychological states [63].

**Figure 3:**
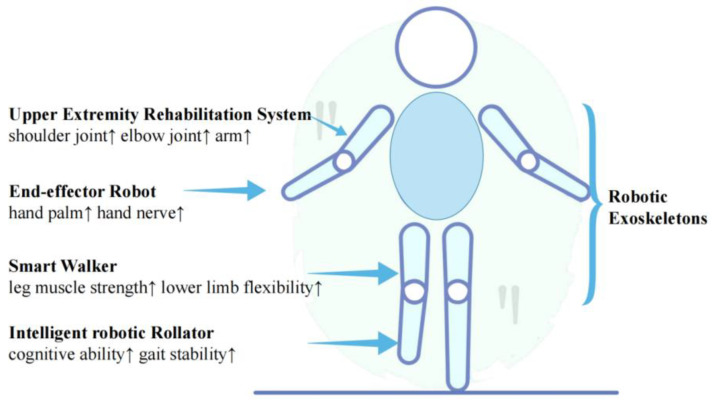
Rehabilitation effects of different types of robots.

Moreover, the study showed that the exercise rehabilitation robot assisted elderly patients with degenerative diseases. It was found that they exhibited a better recovery of their strength and endurance and a significant improvement in their balance and coordination. However, no significant improvement in their agility or speed was observed. The main areas of the body targeted by the different types of rehabilitation robots and their effects are shown in Figure 3.

#### 4.3.1. Better Recovery of Strength and Endurance

Strength is the basis of all physical activities, and the elderly generally show a gradual weakening of muscle strength as they age. Resistance training is the main way to help the elderly restore muscle strength [67]. However, most elderly patients are unable to perform resistance training to improve their strength. The use of the same robotic assistance that is used for sports rehabilitation both improves the elderly’s upper-limb strength and reduces the sports injuries [68] sustained by them [38,39]. This is because these robots can assist the elderly in their progressive resistance training, while monitoring the changes in their physical status during exercise in real-time [20,40]. As shown by their better strength recovery using these methods, compared to traditional recovery training, elderly patients who cannot exercise on their own can improve their muscle strength with the help of these robots [41,69]. The sports rehabilitation robot was found to perform even better in enhancing the endurance of elderly patients. Traditional rehabilitation training is not comparable to robots in terms of the calculation and the control of the load, and the rehabilitation effects of these traditional methods are, thus, not satisfactory [17]. Robotic-assisted gait training was shown to significantly improve the endurance of the elderly patients, compared to traditional treadmill training. Furthermore, the unassisted walking endurance and stair-climbing ability of the elderly patients were also seen to improve [28].

#### 4.3.2. Balance and Coordination Improve the Effect Significantly

Balance and gait disturbances are common manifestations of dysfunction in older adults as they age. Professionals need to accompany elderly patients during the exercises necessary for their balance and coordination training, in order to reduce or avoid falls and sports injuries. This requires a significant amount of human and material resources [70]. In contrast, the use of a sports rehabilitation robot can effectively solve the above problems—the higher the involvement of the sports rehabilitation robot, the more effective it will be in improving the ability of the elderly patients to balance, and, thus, the recovery will be much more effective than when using the non-robotic traditional training methods [66]. In addition, studies have also found that rehabilitation training with sports rehabilitation robots can improve the efficiency of elderly patients’ balance and coordination recovery, can reduce their rehabilitation time, and can have a better effect on their balance function [42,43,44].

#### 4.3.3. No Significant Improvement in Agility and Speed

Agility is a unique physical ability that has not received much attention from rehabilitators, and, thus, there are very few agility training methods for improving the agility of older patients [71]. To some extent, sports rehabilitation robots can be used as training aids to help older adults improve their agility. When combined with technologies such as virtual reality, sports rehabilitation robots can substantially improve the cognitive ability and physical state of elderly patients; however, they cannot significantly improve elderly patients’ performance in agility-related tests [45]. The sports rehabilitation robot also had limited success in improving the speed of the elderly patients. For example, after using robot-assisted gait training, there was no significant difference observed in the improvement of the elderly patients’ gait speed, compared to the traditional treadmill training, and, in some conditions, treadmill training was even seen to yield better results [46]. As a result, there was no significant improvement in the agility and speed of the elderly patients, when they were aided by a sports rehabilitation robot.

## 5. Optimization Suggestions and Future Perspectives

Currently, sports rehabilitation robots use clinical measurement scales to assess whether patients’ functional improvement is significant after receiving the intervention. However, due to the different nature of the ordinal scales used for taking the measurements and their lack of sensitivity in detecting subtle changes in the patient’s exercise performance during the assessments, future studies will need to use specific instruments—such as electromyography and kinematic analysis—to accurately assess the effects of the interventions on the exercise performance and on the motor unit recruitment [72]. Because of the characteristics of the current types of sports rehabilitation robots, their working methods, their effects, and the gaps in the current research, we make the following suggestions.

### 5.1. Refining Assistive Technology

Studies have confirmed that patients with physical dysfunction can recover considerably with the aid of a sports rehabilitation robot [47]. This study found and collated the significant effects that this assistive technology had in improving the physical abilities of elderly patients, such as their strength, endurance, balance, and coordination. However, the current motor rehabilitation assistive technology for neurological and cognitive recovery in elderly patients is inadequate. In the future development of robot-assisted rehabilitation technology, we must focus on the neurological function and cognitive recovery of the patients, based on the recovery of their body function, so that patients can truly recover.

### 5.2. Classification and Characteristics of Sports Rehabilitation Robots

Existing sports rehabilitation robots can provide high-intensity, repeatable rehabilitation training for patients, which can greatly relieve the pressure on staff input in rehabilitation facilities, and can reduce the workload of clinical rehabilitation practitioners. However, compared to professional rehabilitation physicians, sports rehabilitation robots are not able to accurately determine a patient’s recovery or their motivation to recover, based on the patient’s response and physical state, nor are they able to adjust the rehabilitation arrangements in a targeted manner. It is suggested that future sports rehabilitation robots should combine modern multimedia interactive technology to stimulate patients’ interest and enthusiasm in participating in rehabilitation training; to mobilize their awareness and ability to actively participate in training; and to combine multiple modal sensor technologies to achieve intelligent recognition of patients’ physical status and intentions, while having the ability to adjust the rehabilitation plans in a targeted manner.

### 5.3. Enhancing the User Experience

The service object of a sports rehabilitation robot is a human being, and its user experience determines its value. Because the design of the sports rehabilitation robots mainly uses a mechanical multi-link structure, manufacturing is based mainly on alloys, carbon fiber, and other rigid materials. This results in a poor wearing experience for the user, as the prolonged use of a fixed mechanical structure to carry out activities affects the flexibility of the other joints, making it easier for them to sustain sports injuries. It is hoped that the future development of sports rehabilitation robots will include flexible materials to improve the user’s comfort when wearing the devices, based on considerations of safety and support.

## 6. Conclusions

Robot-assisted technology is of great significance for the rehabilitation of the elderly. Sports rehabilitation robots can assist in the enhancement of the physical ability of elderly patients with degenerative diseases, thus, their development and application will have a significant practical value.

The current types of SRR are generally classified as end-effector robots, smart walkers, intelligent robot rollators, and exoskeleton robots—exoskeleton robots are the most widely used. The working methods of these SRR include the following: (1) assistant tools as the main intermediaries—e.g., the robots assist the patients in participating with their rehabilitation; (2) patients as the main intermediaries—e.g., patients dominate the assistant tools to proactively participate in their rehabilitation; and (3) sensors as the main intermediaries—e.g., myoelectric-driven robots promote patient participation. The robot-assisted rehabilitation method is better than the traditional single-motion recovery method, and the elderly patients’ strength and endurance can be better restored through this. In addition, their balance and coordination can also be significantly improved. However, there was no significant improvement in the elderly patients’ agility or speed when they were assisted by these robots.

With the continuous innovation of human-computer interactions, the Internet of Things, artificial intelligence, new materials, robot simulation, and other technologies, the sports rehabilitation robot needs to be further improved through the development of assistive technology, intelligent recognition, user perception, and other technologies, to bring new developments into the field of medical rehabilitation [73]. Crucially, this will bring new hope that more elderly patients with degenerative diseases will be able to resume a normal life.

## Figures and Tables

**Table 1:** Characteristics of included studies (*n* = 38) and information about localization of papers findings in this review.

Author	Year	Country	Study Design
Zhenzhong Zhu, et al. [10]	2022	China	Robot design
Neta Shahar, et al. [11]	2019	Poland	Comparative Trial
Guang Feng, et al. [12]	2022	China	Robot design
Feng Lin, et al. [13]	2019	China	Introduction
Laura Fiorini, et al. [14]	2021	Italy	Needs study
Yuichiro Soma, et al. [15]	2022	Japan	Open-label prospective trial
Sergio D. Sierra M, et al. [16]	2019	Colombia	Robot introduction
Marianna Capecci, et al. [17]	2019	Italy	Randomized controlled trial
Mario F. Jiménez, et al. [18]	2019	Brazil	Robot introduction
George Moustris, et al. [19]	2021	Greece	Evaluation Study
Antonio Frisoli, et al. [20]	2022	Italy.	Clinical control study
Dilber Karagozoglu Coskunsu, et al. [21]	2022	Turkey	Randomized controlled study
Yen-Wei Chen, et al. [22]	2022	China	Randomized cross-over trial
Guotao Li, et al. [23]	2022	China	Robot design
Akim Kapsalyamov, et al. [24]	2020	Australia	Introduction
S.K. Hasan, et al. [25]	2022	America	Robot design
Shuo-Hsiu Chang, et al. [26]	2020	America	Case study
Zlatko Lovrenovic, et al. [27]	2018	Canada	Robot design
Chris McGibbon, et al. [28]	2021	Canada	Open-label randomised cross-over design
Rakel Berriozabalgoitia, et al. [29]	2020	Spain,	Randomized Clinical Trial
Irina Galperin, et al. [30]	2020	Israel;	Cross-sectional study
Simon Christensen, et al. [31]	2021	Denmark	Robot design
Rosaria De Luca, et al. [32]	2020	Italy	Pilot study
Qingming Qu, et al. [33]	2021	China	Functional and clinical experiments
Wonho Choi [34]	2022	Korea	Randomized controlled trial
Lizheng Pan, et al. [35]	2019	China	Preliminary study
Peng Suo, et al. [36]	2022	China	Control algorithm design of mirror rehabilitation training
Jonathan C. Mcleod, et al. [37]	2019	Canada	Robot introduction
Bianca Chinembiri, et al. [38]	2020	China	Randomized clinical trial
Marco Franceschini, et al. [39]	2020	Italy	Follow-up study
Sk. Khairul Hasan, et al. [40]	2020	America	Introduction
Fabian Just, er al. [41]	2020	Switzerland	Robot design
Shih-Ching Chen, et al. [42]	2022	China	Comparative Trial
Silvia Giovannini, et al. [43]	2022	Italy	Randomized controlled trial
Na Ri Yun, et al. [44]	2018	Korea	Randomized controlled trial
Alfredo Manuli, et al. [45]	2020	Italy	Randomized controlled trial
Heejae Kim, et al. [46]	2021	Korea	Randomized controlled trial
Roger Gassert, et al. [47]	2018	Switzerland	Robot design

## Data Availability

Not applicable.

## References

[1] Elliott S.J. (2022). Changing geographies of aging on a global scale: The knowledge to action gap. Can. Geogr..

[2] Falck R.S., Davis J.C., Best J.R., Crockett R.A., Liu-Ambrose T. (2019). Impact of exercise training on physical and cognitive function among older adults: A systematic review and meta-analysis. Neurobiol. Aging.

[3] Saftari L.N., Kwon O.S. (2018). Ageing vision and falls: A review. J. Physiol. Anthropol..

[4] Ahn S., Oh J. (2021). Effects of a health-belief-model-based osteoporosis-and fall-prevention program on women at early oldage. Appl. Nurs. Res..

[5] Sharif S.I., Al-Harbi A.B., Al-Shihabi A.M., Al-Daour D.S., Sharif R.S. (2018). Falls in the elderly: Assessment of prevalence and risk factors. Pharm. Pract. (Granada).

[6] Giovannini S., Brau F., Galluzzo V., Santagada D.A., Loreti C., Biscotti L., Bernabei R. (2022). Falls among older adults: Screening, identification, rehabilitation, and management. Appl. Sci..

[7] Tijsen L.M., Derksen E.W., Achterberg W.P., Buijck B.I. (2019). Challenging rehabilitation environment for older patients. Clin. Interv. Aging.

[8] Tang H., Dai Z. (2022). Health care intervention measures for the elderly under the smart sports rehabilitation mode under the background of big data. Wirel. Commun. Mob. Comput..

[9] Shi D., Zhang W., Zhang W., Ding X. (2019). A review on lower limb rehabilitation exoskeleton robots. Chin. J. Mech. Eng..

[10] Zhu Z., Zheng G., Zhang C. (2022). Development and clinical application of robot-assisted technology in traumatic orthopedics. Chin. J. Reparative Reconstr. Surg..

[11] Shahar N., Schwartz I., Portnoy S. (2019). Differences in muscle activity and fatigue of the upper limb between Task-Specific training and robot assisted training among individuals post stroke. J. Biomech..

[12] Feng G., Zhang J., Zuo G., Li M., Jiang D., Yang L. (2022). Dual-modal hybrid control for an upper-limb rehabilitation robot. Machines.

[13] Lin F., Ajay J., Langan J., Cavuoto L., Nwogu I., Subryan H., Xu W. (2019). QM-FOrMS: A portable and cost-effective upper extremity rehabilitation system. Smart Health.

[14] Fiorini L., De Mul M., Fabbricotti I., Limosani R., Vitanza A., D’Onofrio G., Tsui M., Sancarlo D., Giuliani F., Greco A. (2021). Assistive robots to improve the independent living of older persons: Results from a needs study. Disabil. Rehabil. Assist. Technol..

[15] Soma Y., Mutsuzaki H., Yoshioka T., Kubota S., Shimizu Y., Kanamori A., Yamazaki M. (2022). Single-joint hybrid assistive limb in knee rehabilitation after ACL reconstruction: An open-label feasibility and safety trial. Prog. Rehabil. Med..

[16] Sierra M.S.D., Garzón M., Múnera M., Cifuentes C.A. (2019). Human–robot–environment interaction interface for smart walker assisted gait: AGoRA walker. Sensors.

[17] Capecci M., Pournajaf S., Galafate D., Sale P., Le Pera D., Goffredo M., De Pandis M.F., Andrenelli E., Pennacchioni M., Ceravolo M.G. (2019). Clinical effects of robot-assisted gait training and treadmill training for Parkinson’s disease. A randomized controlled trial. Ann. Phys. Rehabil. Med..

[18] Jiménez M.F., Monllor M., Frizera A., Bastos T., Roberti F., Carelli R. (2019). Admittance controller with spatial modulation for assisted locomotion using a smart walker. J. Intell. Robot. Syst..

[19] Moustris G., Kardaris N., Tsiami A., Chalvatzaki G., Koutras P., Dometios A., Mavridis P. (2021). The i-walk lightweight assistive rollator: First evaluation study. Front. Robot. AI.

[20] Frisoli A., Barsotti M., Sotgiu E., Lamola G., Procopio C., Chisari C. (2022). A randomized clinical control study on the efficacy of three-dimensional upper limb robotic exoskeleton training in chronic stroke. J. NeuroEngineering Rehabil..

[21] Coskunsu D.K., Akcay S., Ogul O.E., Akyol D.K., Ozturk N., Zileli F., Tuzun B.B., Krespi Y. (2022). Effects of robotic rehabilitation on recovery of hand functions in acute stroke: A preliminary randomized controlled study. Acta Neurol. Scand..

[22] Chen Y.W., Chiang W.C., Chang C.L., Lo S.M., Wu C.Y. (2022). Comparative effects of EMG-driven robot-assisted therapy versus task-oriented training on motor and daily function in patients with stroke: A randomized cross-over trial. J. NeuroEngineering Rehabil..

[23] Li G., Cheng L., Sun N. (2022). Design, manipulability analysis and optimization of an index finger exoskeleton for stroke rehabilitation. Mech. Mach. Theory.

[24] Kapsalyamov A., Hussain S., Jamwal P.K. (2020). State-of-the-art assistive powered upper limb exoskeletons for elderly. IEEE Access.

[25] Hasan S.K., Dhingra A.K. (2022). Biomechanical design and control of an eight DOF human lower extremity rehabilitation exoskeleton robot. Results Control. Optim..

[26] Chang S.H., Zhu F., Patel N., Afzal T., Kern M., Francisco G.E. (2020). Combining robotic exoskeleton and body weight unweighing technology to promote walking activity in tetraplegia following SCI: A case study. J. Spinal Cord Med..

[27] Lovrenovic Z., Doumit M. (2019). Development and testing of a passive walking assist exoskeleton. Biocybern. Biomed. Eng..

[28] McGibbon C., Sexton A., Gryfe P., Dutta T., Jayaraman A., Deems-Dluhy S., Novak A., Fabara E., Adans-Dester C., Bonato P. (2021). Effect of using of a lower-extremity exoskeleton on disability of people with multiple sclerosis. Disabil. Rehabil. Assist. Technol..

[29] Berriozabalgoitia R., Sanz B., Fraile-Bermúdez A.B., Otxoa E., Yeregui I., Bidaurrazaga-Letona I., Duñabeitia I., Antigüedad A., Domercq M., Irazusta J. (2020). An overground robotic gait training program for people with multiple sclerosis: A protocol for a randomized clinical trial. Front. Med..

[30] Galperin I., Herman T., Assad M., Ganz N., Mirelman A., Giladi N., Hausdorff J.M. (2020). Sensor-based and patient-based assessment of daily-living physical activity in people with parkinson’s disease: Do motor subtypes play a role?. Sensors.

[31] Christensen S., Rafique S., Bai S. (2021). Design of a powered full-body exoskeleton for physical assistance of elderly people. Int. J. Adv. Robot. Syst..

[32] De Luca R., Maresca G., Balletta T., Cannavò A., Leonardi S., Latella D., Calabrò R.S. (2020). Does overground robotic gait training improve non-motor outcomes in patients with chronic stroke? Findings from a pilot study. J. Clin. Neurosci..

[33] Qu Q., Lin Y., He Z., Fu J., Zou F., Jiang Z., Jia J. (2021). The effect of applying robot-assisted task-oriented training using human-robot collaborative interaction force control technology on upper limb function in stroke patients: Preliminary findings. BioMed Res. Int..

[34] Choi W. (2022). Effects of robot-assisted gait training with body weight support on gait and balance in stroke patients. Int. J. Environ. Res. Public Health.

[35] Pan L., Song A., Wang S., Duan S. (2019). Experimental study on upper-limb rehabilitation training of stroke patients based on adaptive task level: A preliminary study. BioMed Res. Int..

[36] Suo P., Zhu X., Wang S., Li M., Yu T., Song C., Ning H., Xin Y. (2022). A training method for a sensor-based exercise rehabilitation robot. J. Sens..

[37] Mcleod J.C., Ward S.J., Hicks A.L. (2019). Evaluation of the Keeogo™ dermoskeleton. Disabil. Rehabil. Assist. Technol..

[38] Chinembiri B., Ming Z., Kai S., Xiu F.Z., Wei C. (2021). The fourier M2 robotic machine combined with occupational therapy on post-stroke upper limb function and independence-related quality of life: A randomized clinical trial. Top. Stroke Rehabil..

[39] Franceschini M., Mazzoleni S., Goffredo M., Pournajaf S., Galafate D., Criscuolo S., Posteraro F. (2020). Upper limb robot-assisted rehabilitation versus physical therapy on subacute stroke patients: A follow-up study. J. Bodyw. Mov. Ther..

[40] Hasan S.K., Dhingra A.K. (2020). State of the art technologies for exoskeleton human lower extremity rehabilitation robots. J. Mechatron Robot.

[41] Just F., Özen Ö., Tortora S., Klamroth-Marganska V., Riener R., Rauter G. (2020). Human arm weight compensation in rehabilitation robotics: Efficacy of three distinct methods. J. Neuroeng. Rehabil..

[42] Chen S.C., Kang J.H., Peng C.W., Hsu C.C., Lin Y.N., Lai C.H. (2022). Adjustable parameters and the effectiveness of adjunct robot-assisted gait training in individuals with chronic stroke. Int. J. Environ. Res. Public Health.

[43] Giovannini S., Iacovelli C., Brau F., Loreti C., Fusco A., Caliandro P., Castelli L. (2022). Robotic-assisted rehabilitation for balance and gait in stroke patients (ROAR-S): Study protocol for a preliminary randomized controlled trial. Trials.

[44] Yun N., Joo M.C., Kim S.C., Kim M.S. (2018). Robot-assisted gait training effectively improved lateropulsion in subacute stroke patients: A single-blinded randomized controlled trial. Eur. J. Phys. Rehabil. Med..

[45] Manuli A., Maggio M.G., Latella D., Cannavò A., Balletta T., De Luca R., Naro A., Calabrò R.S. (2020). Can robotic gait rehabilitation plus virtual reality affect cognitive and behavioural outcomes in patients with chronic stroke? A randomized controlled trial involving three different protocols. J. Stroke Cerebrovasc. Dis..

[46] Kim H., Kim E., Yun S.J., Kang M., Shin H.I., Oh B., Seo H.G. (2022). Robot-assisted gait training with auditory and visual cues in Parkinson’s disease: A randomized controlled trial. Ann. Phys. Rehabil. Med..

[47] Gassert R., Dietz V. (2018). Rehabilitation robots for the treatment of sensorimotor deficits: A neurophysiological perspective. J. NeuroEng. Rehabil..

[48] Martinez-Martin E., Escalona F., Cazorla M. (2020). Socially assistive robots for older adults and people with autism: An overview. Electronics.

[49] Losey D.P., McDonald C.G., Battaglia E., O’Malley M.K. (2018). A review of intent detection, arbitration, and communication aspects of shared control for physical human–robot interaction. Appl. Mech. Rev..

[50] Bove R., Musallam A., Healy B.C., Houtchens M., Glanz B.I., Khoury S., Chitnis T. (2013). No sex-specific difference in disease trajectory in multiple sclerosis patients before and after age 50. BMC Neurol..

[51] Gu Y., Xu Y., Shen Y., Huang H., Liu T., Jin L., Wang J. (2022). A review of hand function rehabilitation systems based on hand motion recognition devices and artificial intelligence. Brain Sci..

[52] Moggio L., de Sire A., Marotta N., Demeco A., Ammendolia A. (2022). Exoskeleton versus end-effector robot-assisted therapy for finger-hand motor recovery in stroke survivors: Systematic review and meta-analysis. Top. Stroke Rehabil..

[53] Lin F., Ajay J., Langan J., Cavuoto L., Nwogu I., Subryan H., Xu W. A portable and cost-effective upper extremity rehabilitation system for individuals with upper limb motor deficits. Proceedings of the 2016 IEEE Wireless Health (WH).

[54] Abbasimoshaei A., Mohammadimoghaddam M., Kern T.A. (2020). Adaptive fuzzy sliding mode controller design for a new hand rehabilitation robot. Proceedings of the 12th International Conference on Human Haptic Sensing and Touch Enabled Computer Applications.

[55] Moshaii A.A., Moghaddam M.M., Niestanak V.D. (2019). Fuzzy sliding mode control of a wearable rehabilitation robot for wrist and finger. Ind. Robot..

[56] Aly A.A.I., Abbasimoshaei A., Kern T.A. (2022). Developing a VR Training Environment for Fingers Rehabilitation. Proceedings of the13th International Conference on Human Haptic Sensing and Touch Enabled Computer Applications (EuroHaptics 2022).

[57] Molteni F., Gasperini G., Cannaviello G., Guanziroli E. (2018). Exoskeleton and end-effector robots for upper and lower limbs rehabilitation: Narrative review. PMR.

[58] Chalvatzaki G., Koutras P., Tsiami A., Tzafestas C.S., Maragos P. (2020). I-Walk intelligent assessment system: Activity, mobility, intention, communication. Proceedings of the European Conference on Computer Vision.

[59] Moustris G., Kardaris N., Tsiami A., Chalvatzaki G., Koutras P., Dometios A., Mavridis P. (2020). The I-walk assistive robot. Proceedings of the 13th International Workshop on Human-Friendly Robotics.

[60] Weber L.M., Stein J. (2018). The use of robots in stroke rehabilitation: A narrative review. NeuroRehabilitation.

[61] Rupal B.S., Rafique S., Singla A., Singla E., Isaksson M., Virk G.S. (2017). Lower-limb exoskeletons: Research trends and regulatory guidelines in medical and non-medical applications. Int. J. Adv. Robot. Syst..

[62] Gorgey A.S. (2018). Robotic exoskeletons: The current pros and cons. World J. Orthop..

[63] Martinek R., Ladrova M., Sidikova M., Jaros R., Behbehani K., Kahankova R., Kawala-Sterniuk A. (2021). Advanced bioelectrical signal processing methods: Past, present, and future approach—Part III: Other biosignals. Sensors.

[64] Ding I.J., Chang Y.J. (2016). On the use of Kinect sensors to design a sport instructor robot for rehabilitation and exercise training of the elderly. Sens. Mater..

[65] Kropielnicka K., Dziubek W., Bulińska K., Stefańska M., Wojcieszczyk-Latos J., Jasiński R., Pilch U., Dąbrowska G., Skórkowska-Telichowska K., Kałka D. (2018). Influence of the physical training on muscle function and walking distance in symptomatic peripheral arterial disease in elderly. BioMed Res. Int..

[66] Rogers C.E., Cordeiro M., Perryman E. (2014). Maintenance of physical function in frail older adults. Nurs. Clin..

[67] Fragala M.S., Cadore E.L., Dorgo S., Izquierdo M., Kraemer W.J., Peterson M.D., Ryan E.D. (2019). Resistance training for older adults: Position statement from the national strength and conditioning association. J. Strength Cond. Res..

[68] Bessler J., Prange-Lasonder G.B., Schaake L., Saenz J.F., Bidard C., Fassi I., Valori M., Lassen A.B., Buurke J.H. (2021). Safety assessment of rehabilitation robots: A review identifying safety skills and current knowledge gaps. Front. Robot. AI.

[69] Der Loos V., Machiel H.F., Reinkensmeyer D.J., Guglielmelli E. (2016). Rehabilitation and health care robotics. Springer Handbook of Robotics.

[70] Osoba M.Y., Rao A.K., Agrawal S.K., Lalwani A.K. (2019). Balance and gait in the elderly: A contemporary review. Laryngoscope Investig. Otolaryngol..

[71] Ogilvie M., Wallen M.P., Talpey S.W. (2021). Agile ageing–A modifiable vital sign to mitigate the risk of falls in older adults?. Med. Hypotheses.

[72] Porciuncula F., Roto A.V., Kumar D., Davis I., Roy S., Walsh C.J., Awad L.N. (2018). Wearable movement sensors for rehabilitation: A focused review of technological and clinical advances. PMR.

[73] Kondratenko Y.P. (2015). Robotics, automation and information systems: Future perspectives and correlation with culture, sport and life science. Decision Making and Knowledge Decision Support Systems.

